# Synthesis and Positive Inotropic Activity of [1,2,4]Triazolo[4,3-*a*] Quinoxaline Derivatives Bearing Substituted Benzylpiperazine and Benzoylpiperazine Moieties

**DOI:** 10.3390/molecules22020273

**Published:** 2017-02-11

**Authors:** Xue-Kun Liu, Long-Xu Ma, Zhi-Yu Wei, Xun Cui, Shi Zhan, Xiu-Mei Yin, Hu-Ri Piao

**Affiliations:** 1 Tonghua Normal University , College of Pharmaceutical and Food Science, Tonghua 134002, China; liuxuekunth@126.com; 2 Key Laboratory of Natural Resources of Changbai Mountain & Functional Molecules, Ministry of Education, College of Pharmacy, Yanbian University, Yanji 133000, China; xuekunliu@sina.com (L.-X.M.); 15981392912@163.com (Z.-Y.W.); 3 College of Medicine, Yanbian University, Yanji 133000, China; cuixun@ybu.edu.cn; 4 State Key Laboratory of Inorganic Synthesis and Preparative Chemistry, College of Chemistry, Jilin University, Changchun 130000, China; yqwang@126.com

**Keywords:** [1,2,4]triazolo[4,3-*a*] quinoxaline, positive inotropic activity, stroke volume, atrium, milrinone

## Abstract

In an attempt to search for more potent positive inotropic agents, two series of [1,2,4]triazolo[4,3-*a*] quinoxaline derivatives bearing substituted benzylpiperazine and benzoylpiperazine moieties were synthesized and their positive inotropic activities evaluated by measuring left atrial stroke volume in isolated rabbit heart preparations. Several compounds showed favorable activities compared with the standard drug, milrinone. Compound **6c** was the most potent agent, with an increased stroke volume of 12.53% ± 0.30% (milrinone: 2.46% ± 0.07%) at 3 × 10^−5^ M. The chronotropic effects of compounds having considerable inotropic effects were also evaluated.

## 1. Introduction

Glycosides such as digoxin are frequently prescribed cardiotonic agents used for the treatment of congestive heart failure (CHF). Unlike other CHF drugs, they do not increase mortality. However, the narrow safety margins associated with the use of digitalis compounds are serious problems because of the high frequency and severity of digitalis intoxication [1]. The discovery of amrinone led to the synthesis of several agents holding promise for CHF treatment as non-sympathomimetic, non-glycoside agents [2]. The phosphodiesterase-inhibiting agent milrinone has vasodilator and inotropic properties. It was approved for the treatment of CHF more than a decade ago. Nevertheless, the significant ventricular arrhythmias and tachycardia associated with elevated levels of cyclic adenosine monophosphate limit the use of milrinone [3] as well as a newer agent, vesnarinone [4,5]. Therefore, newer positive inotropic agents with fewer side effects are needed [6].

**Figure 1:**
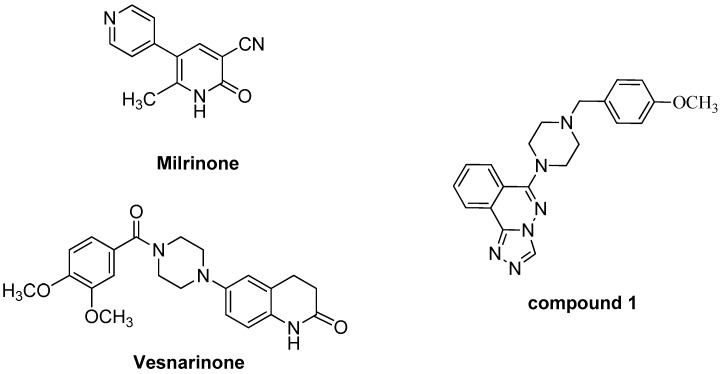
Cardiotonic agents used for the treatment of congestive heart failure (CHF) and the previously reported compound **A**.

Previously, we reported the identification of a [1,2,4]triazolo[3,4-*a*]phthalazine derivative (compound **A**) with remarkable positive inotropic activities that could elicit an increased stroke volume of 9.92% ± 0.09% (Figure 1) [7]. To further optimize compound **A**, based on the principles of bioisosterism, we replaced the [1,2,4]triazolo[3,4-*a*]phthalazine group with a [1,2,4]triazolo[4,3-*a*] quinoxaline group, and simultaneously changed the substituents on the phenyl ring of the benzyl moiety at the 4-position of the piperazine ring, Thus, 11 novel compounds of [1,2,4]triazolo[4,3-*a*] quinoxaline derivatives bearing substituted benzylpiperazine moieties (**6a**–**k**) were synthesized. Moreover, to investigate the effects of benzylpiperazine on activity, we introduced a carbonyl group at the α-position of the benzyl group. Thus, a series of [1,2,4]triazolo[4,3-*a*] quinoxaline derivatives bearing substituted benzoylpiperazine moieties (**7a**–**e**) were also designed and screened for their inotropic activity.

## 2. Results and Discussion

### 2.1. Synthesis

**Scheme 1:**
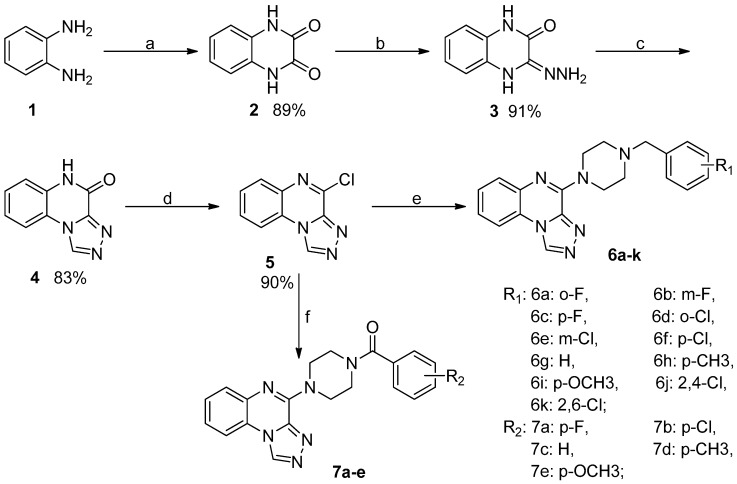
Synthetic scheme for the synthesis of compound **6a**–**k** and **7a**–**e**. Reagents and conditions: (**a**) C_6_H_10_O_4_, reflux, 4 h; (**b**) NH_2_NH_2_H_2_O, reflux, 4 h; (**c**) CH(OC_2_H_5_)_3_, reflux, 3 h; (**d**) POCl_3_, Methylbenzene/DMF, reflux, 3 h; (**e**) monosubstituted piperazines, K2CO3, acetone, reflux, 5 h; (**f**) monosubstituted piperazines, K_2_CO_3_, acetone, reflux, 8 h.

The reaction sequence for the synthesis of **16** new quinoxaline derivatives **6a**–**k** and **7a**–**e** is outlined in Scheme 1. Benzene-1,2-diamine (**1**) was reacted with diethyl oxalate to afford quinoxaline-2,3(1*H*,4*H*)-dione (**2**), which was subsequently reacted with refluxing hydrazine hydrate to give 3-hydrazono-3,4-dihydroquinoxalin-2(1*H*)-one (**3**). Compound **4** was prepared from compound **3** by reaction with ethyl orthoformate. Compound **5** was obtained by reacting **4** with refluxing phosphorus oxychloride (POCl_3_). The nucleophilic aromatic substitution reactions of **5** with various monosubstituted piperazines in refluxing acetone in the presence of potassium carbonate afforded compounds **6a**–**k**. Finally, compounds **7a**–**e** were obtained in high yield from the reactions of **5** with appropriate different monosubstituted piperazines in refluxing acetone in the presence of potassium carbonate. Newly synthesized derivatives **6a**–**k** and **7a**–**e** were characterized by ^1^H-NMR, ^13^C-NMR, IR and mass spectral data. In general, IR spectral data in the ranges of 1549–1457 cm^−1^ and 1638–1620 cm^−1^ indicated distinctive functional groups such as −C=N and −C=O stand for derivatives **6a**–**k** and **7a**–**e**, respectively. The (M + 1) peaks in the mass spectra for these compounds were in agreement with their molecular formula.

### 2.2. Biological Evaluation

Seven of the 16 compounds tested displayed inotropic effects against isolated rabbit heart preparations (Table 1). Compounds **6c**, **6g**, **6h**, and **7c** exhibited more potent effects compared with milrinone (2.46% ± 0.3% at 3 × 10^−5^ M), among which compound **6c** showed the most potent activity, with an increased stroke volume of 12.53% ± 0.30%. For compounds **6a**–**k**, different substituents on the phenyl ring of the benzyl group at the 4-position of the piperazine ring exerted considerable influence on the inotropic activity. For fluorinated compounds, only para-substituted **6c** showed good activity, and clearly exhibited more potent effects compared with lead compound **1** and milrinone, with an increased stroke volume of 12.53% ± 0.30%. Chloro-substituted compounds (**6d**, **6e**, **6j** and **6k**) did not show any inotropic activity, and the para-chloro-substituted **6f** displayed slightly increased activity with an increased stroke volume of 1.01% ± 0.06%. The position of the substituents on the phenyl ring also influenced activity but, in general, a clear pattern for the structure-activity relationship was not found.

In the series of [4-([1,2,4]triazolo[4,3-*a*]quinoxalin-4-yl)piperazin-1-yl](phenyl)methanone-–bearing substituted benzoylpiperazine moieties (**7a**–**e**), compounds possessing the same substituent group as series **6** showed lower activity than those in series **6**. Compound **7a** displayed slightly increased activity with an increased stroke volume of 0.99% ± 0.06%. Only **7c** exhibited more potent effects compared with milrinone, and increased the stroke volume by 4.71% ± 0.05%.

**Figure 2:**
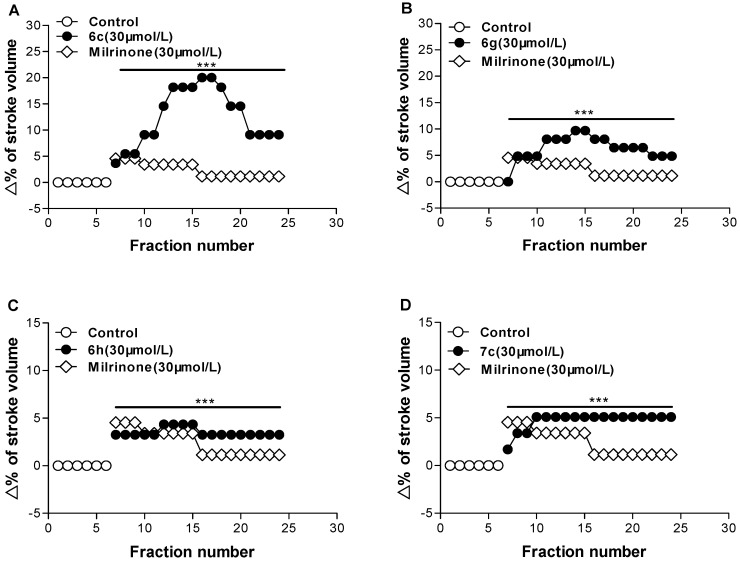
Effects of milrinone and compounds **6c**, **6g**, **6h**, and **7c** on stroke volume in beating rabbit atria (1.5 Hz). Values are means ± SE. *** *p* < 0.001 vs. control. (**A**): effects of compound **6c** on stroke volume in beating rabbit atria, (**B**): effects of compound **6g** on stroke volume in beating rabbit atria, (**C**): effects of compound **6h** on stroke volume in beating rabbit atria, (**D**): effects of compound **7c** on stroke volume in beating rabbit atria.

We investigated the dynamics of the tested compounds in perfused beating rabbit atria. Compounds **6c**, **6g** and **6h** produced initial increases in stroke volume, whereas longer treatment caused decreases in stroke volume (Figure 2A–C). For compound **7c**, the stroke volume increased gradually (Figure 2D).

**Figure 3:**
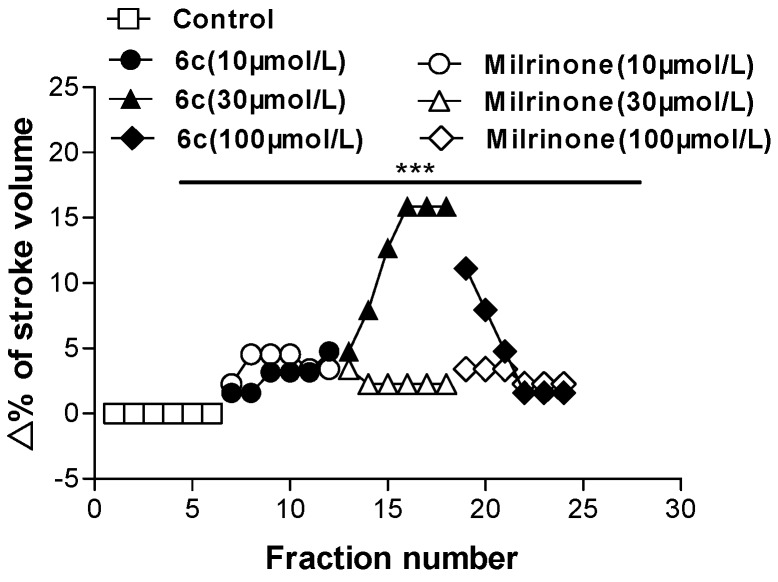
Concentration-response curves of compounds **6c** and milrinone on stroke volume in beating rabbit atria (1.5 Hz). Values are means ± SE. *** *p* < 0.001 versus control.

Next, we tested the dose dependency of the most effective compound (**6c**) at 1 × 10^−5^ M, 3 × 10^−5^ M and 1 × 10^−4^ M. This compound showed maximal effects at 3 × 10^−5^ M, and lower activity at the highest dose (1 × 10^−4^ M) (Figure 3).

## 3. Experimental Section

### 3.1. General Information

Melting points were determined in open capillary tubes and were uncorrected. Chemical reactions were monitored by thin-layer chromatography on silica gel precoated F254 plates (Merck, Whitehouse Station, NJ, USA). Developed plates were visualized by ultraviolet light (254 nm). Column chromatography was undertaken with 200-mesh silica gel (Merck). IR spectra were recorded (in KBr) on a FT-IR 1730 system. ^1^H-NMR spectra were measured on an AV-300 Spectrometer (Bruker, Billerica, MA, USA) using trimethylsilane as an internal standard. Mass spectra were measured on an HP1100LC system (Agilent Technologies, Santa Clara, CA, USA). Chemicals were purchased from Sigma–Aldrich (Saint Louis, MO, USA) and Fluka (Milwaukee, WI, USA).

### 3.2. Synthesis

#### 3.2.1. General Experimental Procedure for the Synthesis of [1,2,4]Triazolo[4,3-*a*] Quinoxaline–Bearing Substituted Benzylpiperazine Moieties (**6a**–**k**)

Compounds **1**–**5** were synthesized by the previously described method [8,9,10,11,12]. A mixture of **5** (0.20 g, 1.0 mmol), monosubstituted piperazine (2.0 mmol) and anhydrous potassium carbonate in acetone was heated at reflux with stirring for 5 h. The solvent was evaporated under reduced pressure, and the resulting residue dissolved in dichloromethane (DCM). The DCM solution was washed sequentially with water and brine, dried over MgSO_4_, and distilled to dryness under reduced pressure. The resulting residue was purified by silica gel column chromatography with DCM and methanol (30:1). The yield, melting point, and spectral data of each compound were recorded.

*4-(4-(2-Fluorobenzyl)piperazin-1-yl)-[1,2,4]triazolo[4,3-a] quinoxaline* (**6a**). Yield: 83%; m.p. 160–164 °C. IR (KBr) cm^–1^: 1542, 1508, 1474 (C=N). ^1^H-NMR (CDCl_3_, 300 MHz, ppm): 2.62–2.63 (m, 4H, (CH_2_)_2_), 3.49–3.55 (m, 4H, (CH_2_)_2_), 4.46 (s, 2H, CH_2_), 7.27–7.73 (m, 8H, Ar-H), 9.17 (s, 1H, N=CH). ^13^C-NMR (CDCl_3_, 75 MHz, ppm): δ 163.08, 159.82, 147.38, 142.34, 138.56, 135.98, 131.68, 129.05, 127.90, 126.92, 123.94, 123.65, 121.60, 115.20, 114.93, 55.31, 52.98, 46.19; MS *m*/*z*: 363 (M + 1).

*4-(4-(3-Fluorobenzyl)piperazin-1-yl)-[1,2,4]triazolo [4,3-a]quinoxaline* (**6b**). Yield: 76%; m.p. 146–150 °C. IR (KBr) cm^–1^: 1543, 1512, 1483 (C=N). ^1^H-NMR (CDCl_3_, 300 MHz, ppm): 2.61–2.64 (m, 4H, (CH_2_)_2_), 3.47–3.55 (m, 4H, (CH_2_)_2_), 4.44 (s, 2H, CH_2_), 6.95–7.69 (m, 8H, Ar-H), 9.15 (s, 1H, N=CH). ^13^C-NMR (CDCl_3_, 75 MHz, ppm): δ 164.60, 161.35, 145.67, 140.59, 139.92, 136.90, 135.52, 129.67, 127.90, 126.94, 124.60, 123.70, 121.62, 115.91, 115.63, 62.43, 53.24, 46.20; MS *m*/*z*: 363 (M + 1).

*4-(4-(4-Fluorobenzyl)piperazin-1-yl)-[1,2,4]triazolo[4,3-a] quinoxaline* (**6c**). Yield: 71%; m.p. 156–158 °C. IR (KBr) cm^–1^: 1541, 1512, 1485 (C=N). ^1^H-NMR (CDCl_3_, 300 MHz, ppm): 2.61–2.65 (m, 4H, (CH_2_)_2_), 3.49–3.55 (m, 4H, (CH_2_)_2_), 4.46 (s, 2H, CH_2_), 7.00–7.73 (m, 8H, Ar-H), 9.17 (s, 1H, N=CH). ^13^C-NMR (CDCl_3_, 75 MHz, ppm): δ 145.73, 139.90, 138.08, 137.03, 135.40, 133.63, 130.75, 129.03, 127.90, 127.00, 124.62, 121.60, 114.65, 63.43, 52.91, 46.19; MS *m*/*z*: 363 (M + 1).

*4-(4-(2-Chlorobenzyl)piperazin-1-yl)-[1,2,4]triazolo[4,3-a] quinoxaline* (**6d**). Yield: 72%; m.p. 178–180 °C. IR (KBr) cm^–1^: 1549, 1508, 1479 (C=N). ^1^H-NMR (CDCl_3_, 300 MHz, ppm): 2.72–2.75 (m, 4H, (CH_2_)_2_), 3.43–3.72 (m, 4H, (CH_2_)_2_), 4.47 (s, 2H, CH_2_), 7.22–7.74 (m, 8H, Ar-H), 9.20 (s, 1H, N=CH). ^13^C-NMR (CDCl_3_, 75 MHz, ppm): δ 145.69, 139.94, 136.91, 135.56, 135.28, 134.47, 130.93, 129.55, 128.40, 128.00, 126.95, 126.68, 122.72, 121.60, 111.52, 53.23, 50.66, 46.21; MS *m*/*z*: 379 (M + 1).

*4-(4-(3-Chlorobenzyl)piperazin-1-yl)-[1,2,4]triazolo[4,3-a] quinoxaline* (**6e**). Yield: 61%; m.p. 160–162 °C. IR (KBr) cm^–1^: 1548, 1511, 1474 (C=N). ^1^H-NMR (CDCl_3_, 300 MHz, ppm): 2.62–2.65 (m, 4H, (CH_2_)_2_), 3.47–3.54 (m, 4H, (CH_2_)_2_), 4.45 (s, 2H, CH_2_), 7.19–7.71 (m, 8H, Ar-H), 9.17 (s, 1H, N=CH). ^13^C-NMR (CDCl_3_, 75 MHz, ppm): δ 145.66, 140.94, 139.96, 136.88, 135.55, 134.25, 134.13, 129.59, 129.47, 129.12, 127.94, 127.43, 126.95, 123.76, 121.61, 52.91, 50.60, 46.17; MS *m*/*z*: 379 (M + 1).

*4-(4-(4-Chlorobenzyl)piperazin-1-yl)-[1,2,4]triazolo[4,3-a] quinoxaline* (**6f**). Yield: 80%; m.p. 162–164 °C. IR (KBr) cm^–1^: 1541, 1512, 1485 (C=N). ^1^H-NMR (CDCl_3_, 300 MHz, ppm): 2.62–2.65 (m, 4H, (CH_2_)_2_), 3.49–3.55 (m, 4H, (CH_2_)_2_), 4.46 (s, 2H, CH_2_), 7.27–7.73 (m, 8H, Ar-H), 9.17 (s, 1H, N=CH). ^13^C-NMR (CDCl_3_, 75 MHz, ppm): δ 145.70, 139.96, 137.28, 136.93, 135.50, 133.00, 130.75, 129.03, 127.97, 127.00, 124.26, 121.65, 114.50, 63.46, 53.19, 46.16; MS *m*/*z*: 379 (M + 1).

*4-(4-Benzylpiperazin-1-yl)-[1,2,4]triazolo[4,3-a] quinoxaline* (**6g**). Yield: 89%; m.p. 150–152 °C. IR (KBr) cm^–1^: 1548, 1517, 1467 (C=N). ^1^H-NMR (CDCl_3_, 300 MHz, ppm): 2.63–2.66 (m, 4H, (CH_2_)_2_), 3.48–3.58 (m, 4H, (CH_2_)_2_), 4.46 (s, 2H, CH_2_), 7.26–7.70 (m, 8H, Ar-H), 9.15 (s, 1H, N=CH). ^13^C-NMR (CDCl_3_, 75 MHz, ppm): δ 145.71, 139.85, 137.71, 136.98, 135.50, 129.26, 128.21, 128.11, 127.24, 126.97, 123.67, 121.62, 114.48, 63.08, 53.27, 46.24; MS *m*/*z*: 345 (M + 1).

*4-(4-(4-Methylbenzyl)piperazin-1-yl)-[1,2,4]triazolo[4,3-a] quinoxaline* (**6h**). Yield: 76%; m.p. 156–158 °C. IR (KBr) cm^–1^: 1549, 1506, 1483 (C=N). ^1^H-NMR (CDCl_3_, 300 MHz, ppm): 2.32–2.35 (m, 3H, CH_3_), 2.61–2.74 (m, 4H, (CH_2_)_2_), 3.32–3.53 (m, 4H, (CH_2_)_2_), 4.45 (s, 2H, CH_2_), 7.08–7.62 (m, 8H, Ar-H), 9.11 (s, 1H, N=CH). ^13^C-NMR (CDCl_3_, 75 MHz, ppm): δ 145.62, 139.87, 136.91, 136.77, 136.49, 135.57, 135.07, 134.65, 128.88, 127.08, 126.86, 123.54, 121.60, 62.81, 53.24, 46.25, 21.16; MS *m*/*z*: 359 (M + 1).

*4-(4-(4-Methoxybenzyl)piperazin-1-yl)-[1,2,4]triazolo[4,3-a] quinoxaline* (**6i**). Yield: 66%; m.p. 154–156 °C. IR (KBr) cm^–1^: 1549, 1513, 1479 (C=N). ^1^H-NMR (CDCl_3_, 300 MHz, ppm): 2.45–2.59 (m, 4H, (CH_2_)_2_), 3.44–3.49 (m, 4H, (CH_2_)_2_), 3.81 (s, 2H, CH_2_), 4.41 (s, 3H, CH_3_), 7.27–7.73 (m, 8H, Ar-H), 9.17 (s, 1H, N=CH). ^13^C-NMR (CDCl_3_, 75 MHz, ppm): δ 158.82, 145.61, 139.87, 136.88, 135.55, 130.43, 129.63, 127.83, 126.83, 123.57, 121.56, 114.48, 113.54, 62.41, 55.25, 53.12, 46.16; MS *m*/*z*: 375 (M + 1).

*4-(4-(2,4-Dichlorobenzyl)piperazin-1-yl)-[1,2,4]triazolo[4,3-a] quinoxaline* (**6j**). Yield: 59%; m.p. 180–182 °C. IR (KBr) cm^–1^: 1545, 1508, 1469 (C=N). ^1^H-NMR (CDCl_3_, 300 MHz, ppm): 2.60–2.77 (m, 4H, (CH_2_)_2_), 3.47–3.82 (m, 4H, (CH_2_)_2_), 4.42 (s, 2H, CH_2_), 7.14–7.72 (m, 7H, Ar-H), 9.16 (s, 1H, N=CH). ^13^C-NMR (CDCl_3_, 75 MHz, ppm): δ 145.58, 139.70, 136.65, 135.67, 134.82, 134.45, 133.17, 132.74, 131.77, 131.63, 128.90, 127.48, 126.92, 123.84, 121.90, 55.19, 53.85, 49.36; MS *m*/*z*: 413 (M + 1).

*4-(4-(2,6-Dichlorobenzyl)piperazin-1-yl)-[1,2,4]triazolo[4,3-a] quinoxaline* (**6k**). Yield: 53%; m.p. 198–200 °C. IR (KBr) cm^–1^: 1549, 1510, 1483 (C=N). ^1^H-NMR (CDCl_3_, 300 MHz, ppm): 2.62–2.77 (m, 4H, (CH_2_)_2_), 3.47–3.79 (m, 4H, (CH_2_)_2_), 4.46 (s, 2H, CH_2_), 7.21–7.70 (m, 7H, Ar-H), 9.17 (s, 1H, N=CH). ^13^C-NMR (CDCl_3_, 75 MHz, ppm): δ 145.62, 139.99, 137.04, 135.50, 133.99, 130.94, 129.00, 128.83, 128.43, 127.95, 123.63, 121.60, 114.48, 56.45, 53.20, 50.83; MS *m*/*z*: 413 (M + 1).

#### 3.2.2. [1,2,4]triazolo[4,3-a] quinoxaline Derivatives Bearing Substituted Benzoylpiperazine Moieties (**7a**–**e**)

A mixture of **5** (0.20 g, 1.0 mmol), monosubstituted piperazine (2.0 mmol) and anhydrous potassium carbonate in acetone was heated at reflux with stirring for 8 h. The solvent was evaporated under reduced pressure, and the resulting residue dissolved in DCM. The DCM solution was washed sequentially with water and brine, dried over MgSO_4_, and distilled to dryness under reduced pressure. The resulting residue was purified by silica gel column chromatography with DCM and methanol (20:1). The yield, melting point, and spectral data of each compound were recorded.

*(4-([1,2,4]Triazolo[4,3-a]quinoxalin-4-yl)piperazin-1-yl)(4-fluorophenyl)methanone* (**7a**). Yield: 57%; m.p.: 170–172 °C; IR (KBr) cm^–1^: 1638 (C=O), 1540, 1519, 1459 (C=N) ; ^1^H-NMR (CDCl_3_, 300 MHz, ppm): δ 3.59–3.66 (m, 4H, (CH_2_)_2_), 3.81–4.37 (m, 4H, (CH_2_)_2_), 7.01–7.68 (m, 8H, Ar-H), 9.15 (s, 1H, N=CH). ^13^C-NMR (CDCl_3_, 75 MHz, ppm): δ 169.72, 165.18, 160.69, 145.49, 139.64, 137.28, 136.13, 131.36, 128.05, 127.11, 124.43, 121.75, 115.27, 114.70, 53.50, 50.55; MS *m*/*z*: 377 (M + 1).

*(4-([1,2,4]Triazolo[4,3-a]quinoxalin-4-yl)piperazin-1-yl)(4-chlorophenyl)methanone* (**7b**). Yield: 55%; m.p.: 176–178 °C; IR (KBr) cm^–1^: 1630 (C=O), 1544, 1516, 1457 (C=N) ; ^1^H-NMR (CDCl_3_, 300 MHz, ppm): δ 3.71–3.98 (m, 4H, (CH_2_)_2_), 4.50–5.30 (m, 4H, (CH_2_)_2_), 7.27–7.78 (m, 8H, Ar-H), 9.22 (s, 1H, N=CH). ^13^C-NMR (CDCl_3_, 75 MHz, ppm): δ 169.59, 145.55, 139.75, 136.36, 136.18, 135.96, 133.67, 128.93, 128.74, 128.18, 127.21, 124.57, 121.79, 114.64, 56.04, 50.55; MS *m*/*z*: 393 (M + 1).

*(4-([1,2,4]Triazolo[4,3-a]quinoxalin-4-yl)piperazin-1-yl)(phenyl)methanone* (**7c**). Yield: 76%; m.p.: 216–218 °C; IR (KBr) cm^–1^: 1625 (C=O), 1544, 1508, 1460 (C=N) ; ^1^H-NMR (CDCl_3_, 300 MHz, ppm): δ 3.51–3.69 (m, 4H, (CH_2_)_2_), 3.99–4.49 (m, 4H, (CH_2_)_2_), 7.09–7.76 (m, 9H, Ar-H), 9.20 (s, 1H, N=CH). ^13^C-NMR (CDCl_3_, 75 MHz, ppm): δ 170.68, 146.25, 139.78, 137.62, 135.67, 135.39, 130.04, 129.54, 128.64, 127.23, 127.14, 124.45, 121.80, 114.63, 52.74, 48.83; MS *m*/*z*: 359 (M + 1).

*(4-([1,2,4]Triazolo[4,3-a]quinoxalin-4-yl)piperazin-1-yl)(p-tolyl)methanone* (**7d**). Yield: 71%; m.p.: 168–170 °C; IR (KBr) cm^–1^: 1624 (C=O), 1544, 1516, 1458 (C=N) ; ^1^H-NMR (CDCl_3_, 300 MHz, ppm): δ 2.41 (s, 3H, CH3), 3.48–3.53 (m, 4H, (CH_2_)_2_), 3.76–3.95 (m, 4H, (CH_2_)_2_), 7.23–7.71 (m, 8H, Ar-H), 9.21 (s, 1H, N=CH). ^13^C-NMR (CDCl_3_, 75 MHz, ppm): δ 169.99, 147.11, 141.30, 137.43, 136.74, 135.43, 133.67, 128.94, 128.37, 127.39, 126.21, 124.52, 121.94, 114.51, 52.06, 49.73, 21.57; MS *m*/*z*: 373 (M + 1).

*(4-([1,2,4]Triazolo[4,3-a]quinoxalin-4-yl)piperazin-1-yl)(4-methoxyphenyl)methanone* (**7e**). Yield: 59%; m.p.: 198–200 °C; IR (KBr) cm^–1^: 1624 (C=O), 1545, 1516, 1460 (C=N) ; ^1^H-NMR (CDCl_3_, 300 MHz, ppm): δ 3.43–3.51 (m, 4H, (CH_2_)_2_), 3.67–3.86 (m, 4H, (CH_2_)_2_), 3.97 (s, 3H, CH_3_), 7.21–7.70 (m, 8H, Ar-H), 9.19 (s, 1H, N=CH). ^13^C-NMR (CDCl_3_, 75 MHz, ppm): δ 170.14, 149.27, 143.30, 139.49, 137.44, 136.79, 134.44, 129.90, 128.00, 127.94, 126.39, 124.82, 121.04, 114.73, 60.21, 53.61, 47.93; MS *m*/*z*: 377 (M + 1).

### 3.3. Pharmacology

The method of measuring stroke volume in the left atrium (LA) has been described [13]. The features of CHF are cardiac dilatation, poor contractility of cardiac muscle, decreased ejection fraction, and depression of left ventricular pressure maximum alleosis. Therefore, macroscopic measurement of the variance in LA stroke volume can be used to estimate the positive inotropic effects of the compounds synthesized. Milrinone (Shuzhou Unite Pharmaceuticals, Shuzhou, China), dimethyl sulfoxide (DMSO; Sigma–Aldrich) were purchased. All other reagents were of analytical grade. Atria were obtained from New Zealand white rabbits, and the mean weight of the LA was 183.6 ± 6.8 mg. Hearts were removed from rabbits and the LA dissected free. A calibrated transparent atrial cannula containing two small catheters was inserted into the LA. The cannulated LA was transferred to an organ chamber and perfused immediately with *N*-2-hydroxyethyl piperazine-*N*-2-ethanesulfonic acid (HEPES) buffer solution by means of a peristaltic pump (1.25 mL/min) at 34 °C. The composition of the buffer was (in mM): 118 NaCl, 4.7 KCl, 2.5 CaCl_2_, 1.2 MgCl_2_, 25 NaHCO_3_, 10.0 glucose, 10.0 HEPES (adjusted to pH 7.4 with 1 M NaOH) and 0.1% bovine serum albumin. Soon after the perfusion system was set up, transmural electrical field stimulation with a luminal electrode was started at 1.5 Hz (duration, 0.3–0.5 ms; voltage, 30 V). Changes in LA stroke volume were monitored by reading the lowest level of the water column in the calibrated atrial cannula during end diastole. Atria were perfused for 60 min to stabilize the stroke volume. The atrial beat rate was fixed at 1.5 Hz, LA stroke volume recorded at 2 min intervals, and the stimulus effect of the sample recorded after one circulation in the control group. Each circulation was 12 min. Compounds were investigated using the single-dose method at 3 × 10^−5^ M. Samples were dissolved in DMSO and diluted with HEPES buffer to 0.1% DMSO. Biological data for these compounds were expressed in mean percentage values of increased stroke volume (Table 1). Heart-rate measurements for selected compounds were carried out in isolated rabbit hearts by recording the electrocardiogram in the volume conduction model. To assess differences, repeated measurements were compared by an ANOVA test followed by Bonferroni’s multiple-comparison test. *p* < 0.05 was considered significant and data are the mean ± SE.

## 4. Conclusions

Two series of [1,2,4]triazolo[4,3-*a*] quinoxaline derivatives bearing substituted benzylpiperazine and benzoylpiperazine moieties were synthesized using **A** as the lead compound. We tried to ascertain potent compounds for cardiac contractility without increasing the heart rate. Compound **6c** exhibited promising cardiovascular properties and potent activities compared with milrinone: **6c** was 5.1-fold more active than milrinone. This compound is undergoing further biological tests, including in vivo evaluation, coronary vasodilation tests, and studies into possible mechanisms of action.

## Figures and Tables

**Table 1:**
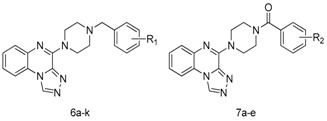
Positive inotropic activity of the test compounds.

Compound	R	Increased Stroke Volume (%) ^a^
**6a**	*o*-F	— ^b^
**6b**	*m*-F	—
**6c**	*p*-F	12.53 ± 0.30
**6d**	*o*-Cl	—
**6e**	*m*-Cl	—
**6f**	*p*-Cl	1.01 ± 0.06
**6g**	H	6.36 ± 0.13
**6h**	*p*-CH_3_	3.50 ± 0.03
**6i**	*p*-OCH_3_	0.63 ± 0.05
**6j**	2,4-Cl	—
**6k**	2,6-Cl	—
**7a**	*p*-F	0.99 ± 0.06
**7b**	*p*-Cl	—
**7c**	H	4.71 ± 0.05
**7d**	*p*-CH_3_	—
**A**	—	9.92 ± 0.09
milrinone		2.46 ± 0.07

^a^ The concentration for the test sample is 3 × 10^−5^ M. ^b^ None or negative stroke volume increase.
